# Supplementary material for: Multimodal imaging and functional analysis of the chick NMDA retinal damage model
Source: PLoS One. 2021 Sep 7;16(9):e0257148. doi: 10.1371/journal.pone.0257148 (PMC8423281; doi:10.1371/journal.pone.0257148)
Supplement: S1 File — (ZIP) [file pone.0257148.s004.zip › SD-OCT/C200727/009/D1-PWk1/C200727-009_OS_V_14X14_0_REGAVG0000009/Manual_PreDefineFiveByFiveGridRectangularRetina_1.pdf]

# Five By Five Grid Template Report

| | | | |
|---|---|---|---|
| **Patient ID** | C200727 | **Name** | See InVivoVue |
| **Exam Date** | See InVivoVue | **Exam Time** | See InVivoVue |
| **Eye** | OS | **Averaged** | N/A |
| **Depth (mm)** | 1.65 | **Depth Samples** | 1024 |
| **Pitch (um/pix)** | 35.000 x 35.000 x 1.608 | **Refractive Index** | See InVivoVue |
| **Scan Type** | Rectangular 14.000mm x 14.000mm x 1.647mm , 400 x 400 x 1024 | | |

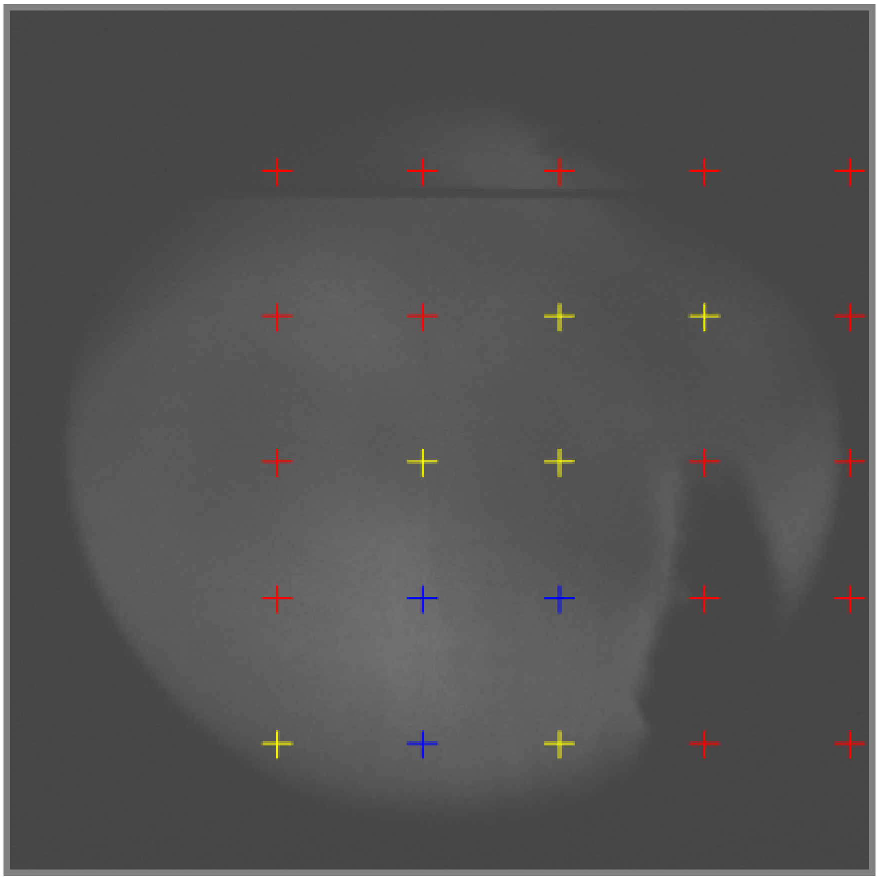

## Thickness Metrics

| Analysis | Average(mm) | Minimum(mm) | Maximum(mm) | STDEV(mm) |
|---|---|---|---|---|
| **IRNFL - RPE** | 0.3224 | 0.2914 | 0.3389 | 0.0269 |
| **IRNFL - ORNFL** | 0.0593 | 0.0412 | 0.0730 | 0.0164 |
| **ORNFL - OPL** | 0.2012 | 0.1928 | 0.2103 | 0.0088 |
| **OPL - ETPRS** | 0.0437 | 0.0356 | 0.0555 | 0.0105 |
